# Use of the SYNTAX Score II to predict mortality in interventional cardiology: A systematic review and meta-analysis

**DOI:** 10.1097/MD.0000000000014043

**Published:** 2019-01-11

**Authors:** Hua Yang, Li Zhang, Chen Hong Xu

**Affiliations:** a Department of Cardiology; b Department of Dermatology, Jingzhou Central Hospital, the Second Clinical Medical College, Yangtze University, Jingzhou, Hubei, China.

**Keywords:** coronary artery disease, mortality, percutaneous coronary intervention, SYNTAX Score II

## Abstract

**Background:**

As the SYNTAX Score has limitations, it should be replaced by another better angiographic tool. By comparing mortality that was observed following percutaneous coronary intervention (PCI) in patients who were allotted a low versus a high score, we aimed to systematically investigate mortality prediction using the SYNTAX Score II in Interventional Cardiology.

**Methods:**

Electronic databases were searched for relevant publications using the terms “SYNTAX Score II and percutaneous coronary intervention.” The main outcome was all-cause mortality. This analysis was carried out by the RevMan 5.3 software [risk ratios (RRs) and 95% confidence intervals (95% CIs) were calculated].

**Results:**

A total number of 9443 participants were enrolled for this analysis. As different studies reported different range of SYNTAX Score II, we further classified these scores range into 4 different groups: 17 < SS > 17, 20 < SS > 20, 22 < SS > 22, and 26 < SS > 26 appropriately. Results of this analysis showed that the risk of mortality in patients with a high SYNTAX Score II (SS > 17) was significantly higher (RR: 2.65, 95% CI: 1.05–6.73; *P* = .04) than patients with a low SYNTAX Score II (SS < 17). Even when participants with a low SYNTAX Score II (SS < 20) were compared with patients who were assigned to a higher SYNTAX Score II (SS > 20), a significantly higher risk of mortality was associated with a high SYNTAX Score II (RR: 3.73, 95% CI: 1.99 – 6.96; *P* = .0001).

**Conclusion:**

Following PCI, the risk of mortality was higher in those patients with a high SYNTAX Score II. The SYNTAX Score II might be considered as an important tool to predict mortality in Interventional Cardiology. Future research should further explore the benefits of this tool.

## 1 Introduction

The SYNTAX score has been developed with reference to the Synergy between percutaneous coronary intervention (PCI) with TAXUS and Cardiac Surgery (SYNTAX) trial, comparing PCI with coronary artery bypass surgery (CABG) in patients with left main and multivessel coronary artery diseases (CADs).^[1]^ It is an important angiographic grading tool that has recently been used clinically to calculate the complexity of CAD. New scientific reports have shown that this scoring system is also capable of predicting major cardiovascular events in patients undergoing PCI.^[2]^ In other words, patients with a high SYNTAX score had lesions that were more complicated and resulted in worst prognosis following PCI.

However, as the SYNTAX score was exclusively dependent only on the anatomical features of abnormal coronary vessels and lesion characteristics (such as the total number of lesions, which were observed, bifurcation or trifurcation lesions, total occlusion, calcification, thrombus formation, aorta-ostial stenosis)^[3]^ without taking into account clinical variables, its use was therefore thought to be limited.

Recently, the SYNTAX Score II, another more sophisticated tool that combined both anatomical and clinical factors together to predict post-procedural outcomes, was developed.^[4]^ Apparently, mortality prediction using this new angiographic tools has seldom been systematically analyzed. Therefore, by comparing mortality that was observed following PCI with a low versus a high score, we aimed to systematically investigate mortality prediction using the SYNTAX Score II in Interventional Cardiology.

## 2 Methods

### 2.1 Searched databases

The following electronic databases were searched for English language publications (between July and August 2018) with reference to the PRISMA guideline^[5]^:

(1) The National Library of Medical Publications (MEDLINE); including its subset PubMed;
(2) EMBASE database (www.sciencedirect.com);
(3) The Cochrane database of Randomized Controlled Trials;
(4) Google Scholar;
(5) Reference lists of relevant articles;
(6) Official websites of cardiovascular journals that aimed at publishing articles, which were related to Interventional Cardiology, such as the *Journal of the American College of Cardiology* (JACC), *Circulation*, *Journal of Cardiology*, *European Heart Journal* (EHJ), and the *International Journal of Cardiology*.

### 2.2 Searched strategy

The searched terms that were used included

(1) SYNTAX Score;
(2) SYNTAX Score II;
(3) Percutaneous coronary intervention and SYNTAX Score II;
(4) Interventional cardiology and SYNTAX Score II;
(5) Drug eluting stents and SYNTAX Score II;
(6) Coronary angioplasty and SYNTAX Score II;
(7) Acute coronary syndrome and SYNTAX Score II.

Abbreviations such as PCI and SS II were also used during this search process.

### 2.3 Inclusion and exclusion criteria

Studies were included if

(1) They were randomized trials or observational studies consisting of patients with CAD undergoing PCI;
(2) They involved patients who were evaluated using the SYNTAX Score II;
(3) They had a control (low score) group and an experimental (high score) group;
(4) They reported mortality that was observed following PCI.

Studies were excluded if:

(1) They were meta-analysis, reviews, case studies, or letters to editors;
(2) They did not involve patients who were treated by PCI;
(3) The SYNTAX Score II was not used to evaluate the patients;
(4) They did not have a control group;
(5) They did not report mortality as their respective clinical outcome;
(6) They did not include relevant data that were applicable to this analysis;
(7) They were duplicated or repeated studies.

### 2.4 Types of participants, outcomes, and follow-up time periods

This analysis consisted only of patients with CAD undergoing PCI (Table 1).^[6–13]^ However, different categories of CAD patients ranging from simple to more complex were included as follows:

(1) Single-vessel CAD;
(2) Two-vessel CAD;
(3) Multivessel CAD;
(4) Unprotected left main CAD (ULMCAD);
(5) STEMI;
(6) Any other complex CAD.

**Table 1:**
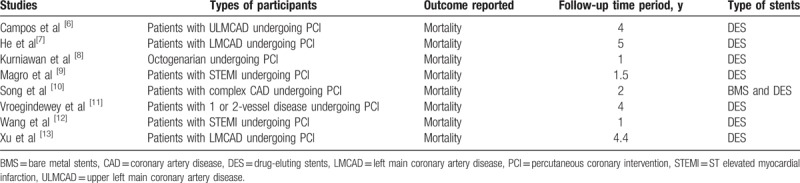
Types of participants, reported outcomes, and follow-up time periods.

The main outcome that was assessed was all-cause mortality.

The follow-up periods varied from study to study, ranging from 1 to 5 years (Table 1).

### 2.5 Data extraction and quality assessment

The following relevant information and data that were associated with the studies were carefully extracted by 3 independent authors:

(1) Authors’ names;
(2) Publication year;
(3) Trial/registry/hospital names;
(4) Methodological quality of the studies;
(5) Baseline features;
(6) Outcomes reported;
(7) Follow-up periods;
(8) Types of participants (single or multivessel CAD, ULMCAD, any other complex CAD);
(9) Location (regions where these studies were carried out);
(10) Type of angiographic tools that were used for evaluation (SS II);
(11) Number of patients with a low versus a high score, respectively.

The methodological qualities of the studies were also assessed. Randomized controlled trials were assessed with reference to the Cochrane Collaboration.^[14]^ Two points were given for each of the 6 components (high risk bias = 0 point, unclear bias = 1 point, low risk of bias = 2) that assessed the methodological quality with a maximum total score of 12 points.

For the observational studies, quality assessment was carried out using the Newcastle–Ottawa Scale (NOS)^[15]^ whereby several components were assessed and “stars” were given to represent the quality of the studies. The maximum total number of stars allotted was 9 (^∗∗∗∗∗∗∗∗∗^).

### 2.6 Statistical analysis

This research article is a systematic review and meta-analysis of previously published studies. Therefore, inconsistency across the studies was obvious because different studies reported different types of patients and data.^[16]^ Heterogeneity across the studies was assessed by

(1) The *Q* statistic test (*P* ≤ .05 was considered statistically significant);
(2) The *I*^2^ statistic test (heterogeneity increased with an increased *I*^2^ value).

In addition, either a fixed effects (*I*^2^ < 50%) model or a random effects (*I*^2^ > 50%) model was used during the subgroup analysis based on the *I*^2^ value that was obtained.

Sensitivity analysis was carried out to ensure that the results were not influenced by one particular study. In addition, publication bias was visually estimated through funnel plots.

The analysis was carried out by the RevMan 5.3 software [risk ratios (RRs) and 95% confidence intervals (95% CIs) were calculated].

### 2.7 Ethical approval

Ethical or board review approval was not required for this meta-analysis.

## 3 Results

### 3.1 Searched outcomes

A total number of 574 publications were obtained through electronic searched databases.

The searched outcomes were as follows:

MEDLINE: 235;

EMBASE: 76;

Cochrane Library: 29;

Reference lists: 15;

Official websites of relevant journals: 22;

Google scholar: 197;

Total number of articles that were obtained: 574.

Direct elimination based upon an assessment of the titles and abstracts: 396.

Full-text articles that were assessed for eligibility: 178.

Further eliminations were based on

(1) duplicated or repeated studies (138);
(2) meta-analysis (2);
(3) letter to editors (2);
(4) involved the SYNTAX score (21);
(5) involved SS II in patients undergoing CABG (3);
(6) Data that could not be used (4).

**Figure 1:**
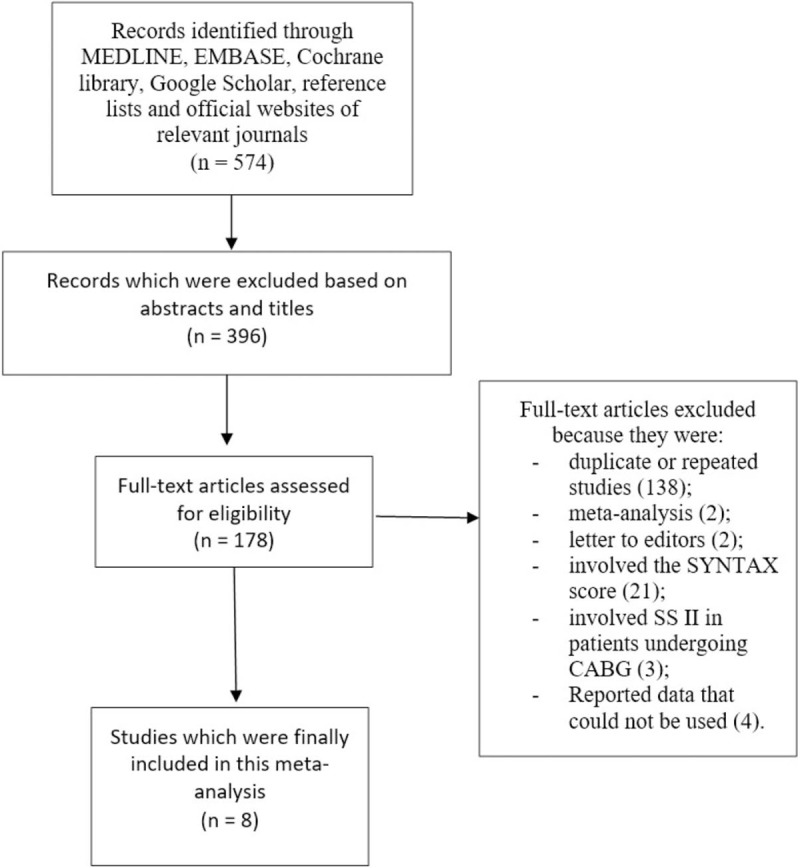
Flow diagram representing the study selection.

Finally, a total number of 8 studies^[6–13]^ were included in this meta-analysis as shown in Fig. 1.

### 3.2 General features of the studies

A total number of 9443 participants were enrolled in this analysis whereby 3633 patients were assigned to a low SYNTAX Score II group and 5810 patients were assigned to a high SYNTAX Score II group. Patients’ enrollment was between the years 2004 and 2014. As different studies reported different range of SYNTAX Score II, we further classified these scores range into 4 different groups: 17 < SS > 17, 20 < SS > 20, 22 < SS > 22, and 26 < SS > 26. These features have been listed in Table 2.

**Table 2:**
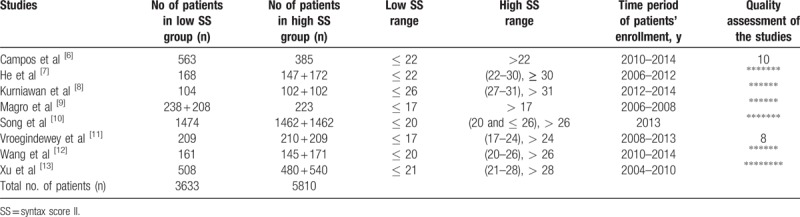
General features of the studies.

### 3.3 Baseline features of the participants

The baseline features of the patients have been listed in Table 3. The mean age of the participants ranged from 50.3 to 82.9 years with a predominance of male patients in this analysis. The other risk factors for cardiovascular diseases such as hypertension, diabetes mellitus, smoker, and dyslipidemia in both groups have been reported in Table 3. No significant difference was observed in baseline features between participants who were assigned a low versus a high SYNTAX Score II.

**Table 3:**
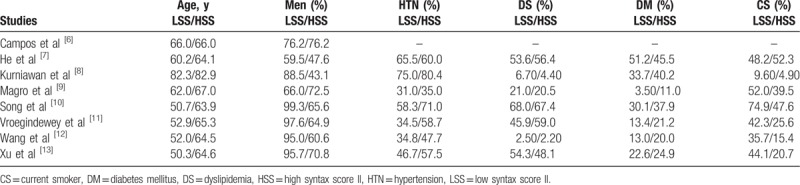
Baseline features of the participants.

### 3.4 Mortality prediction using the SYNTAX Score II (Main result of this analysis)

**Figure 2:**
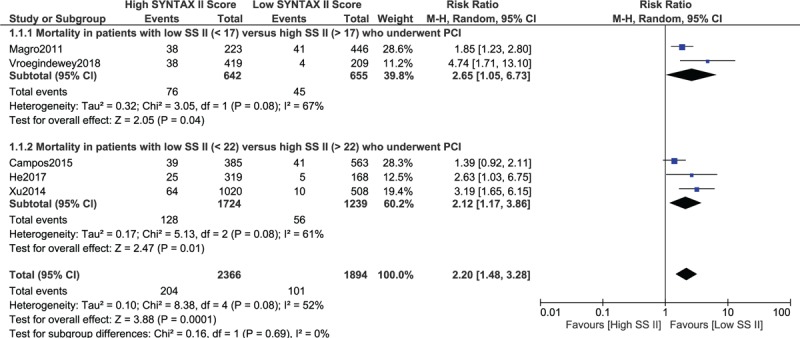
Mortality risk following percutaneous coronary intervention with a low SYNTAX Score II versus a high SYNTAX Score II (Part A).

Results of this analysis showed that the risk of mortality in patients with a high SYNTAX Score II (SS > 17) was significantly higher (RR: 2.65, 95% CI: 1.05 – 6.73; *P* = .04) than patients with a low SYNTAX Score II (SS < 17) as shown in Fig. 2.

**Figure 3:**
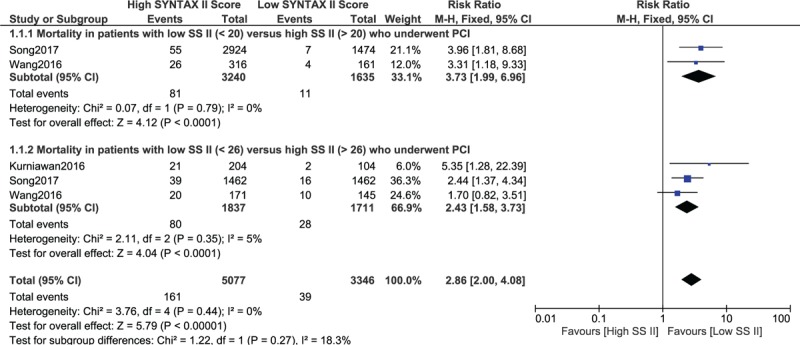
Mortality risk following percutaneous coronary intervention with a low SYNTAX Score II versus a high SYNTAX Score II (Part B).

Even when participants with a low SYNTAX Score II (SS < 20) were compared with patients who were assigned to a higher SYNTAX Score II (SS > 20), a significantly higher risk of mortality was associated with a high SYNTAX Score II (RR: 3.73, 95% CI: 1.99–6.96; *P* = .0001) as shown in Fig. 3.

When participants with a low SYNTAX Score II (SS < 22) were compared with patients who were assigned to a higher score (SS > 22), again the latter was associated with a significantly higher mortality risk (RR: 2.12, 95% CI: 1.17–3.86; *P* = .01) as shown in Fig. 2.

Another analysis was carried out with participants who were assigned to a low (SS < 26) versus high (SS > 26) SYNTAX Score II. Still, a higher SYNTAX Score II predicted a higher risk of mortality following PCI (RR: 2.43, 95% CI: 1.58–3.73; *P* = .001) as shown in Fig. 3.

A summary of the results has been given in Table 4.

**Table 4:**
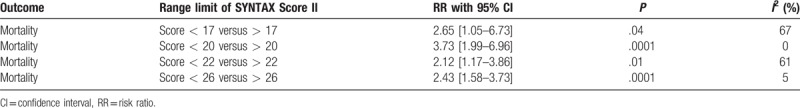
Results of this analysis.

### 3.5 Sensitivity analysis and publication bias

**Figure 4:**
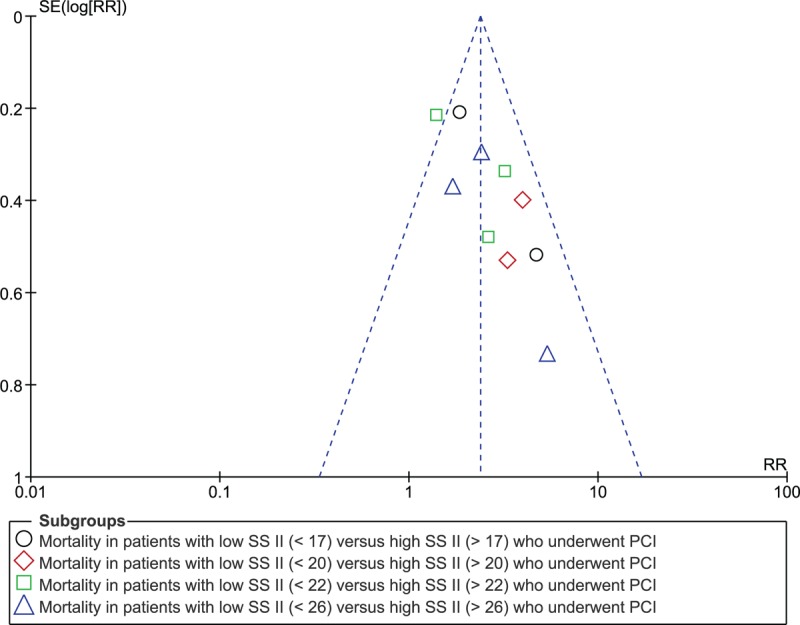
Funnel plot showing publication bias.

Consistent results were obtained throughout when sensitivity analyses were carried out. The results that were obtained when each study was excluded one at a time and a new analysis was carried out were not significantly different as compared to the main results of this analysis. In addition, while observing the funnel plot, we could state that there was only moderate evidence of publication bias among all the studies that assessed mortality after PCI in those patients who were assigned to a low versus a high SYNTAX Score II due to the minor asymmetry of the funnel plot (Fig. 4).

## 4 Discussion

Previously, the SYNTAX score was only used as a guideline for decision making between PCI and CABG. For example, in patients with 3-vessel disease having a low SYNTAX score, PCI was an acceptable choice in comparison to CABG.^[17]^ The SYNTAX score was seldom being used as a prognostic tool in Interventional cardiology. However, newer scientific reports have shown its importance in Interventional cardiology as well,^[18,19]^ but the fact that it also has limitations when compared with newer tools, we were compelled to further consider other tools.^[20]^

The SYNTAX score might not be sufficiently powerful because it only takes into consideration the anatomical and lesion characteristics of the diseased coronary arteries, ignoring the clinical aspects and conditions of the patients. Therefore, other tools that considered the clinical aspects of the patients along with anatomical and lesion characteristics of the coronary arteries, such as the SYNTAX Score II, might replace the SYNTAX score in the future.

According to the results that were obtained from this current analysis, a low SYNTAX Score II was associated with a significantly lower risk of mortality following PCI. The outcome was analyzed after having subdivided the score into different groups with similar range limits. A high SYNTAX Score II could significantly predict mortality following PCI.

Recently, the Evaluation of the Xience Everolimus Eluting Stent versus Coronary Artery Bypass Surgery for Effectiveness of Left Main Revascularization (EXCEL) trial^[21]^ to validate the SYNTAX Score II showed the latter to indicate at least an equipoise for mortality observed between PCI (10.1% vs 7.3%) and CABG (10.8% vs 10.3%) during the long-term [OR between PCI and CABG: 0.79, 95% CI: 0.43–1.50 at 4-years follow-up]. The authors also stated that both the clinical [age, creatinine clearance, left ventricular ejection fraction, left main CAD (LMCAD), female sex, chronic obstructive pulmonary disease, and peripheral vascular diseases (PVDs)] and anatomical (anatomical SYNTAX score) features had a visible impact in predicting long-term mortality and in reaching a decision based on the most beneficial revascularization choice.

A recently published meta-analysis comparing the SYNTAX score with clinical SYNTAX Score to validate their abilities to predict adverse clinical outcomes showed that the latter was associated with better predictive value for all-cause mortality with RR: 1.04 (95% CI: 1.03–1.05).^[22]^ Even in this current analysis, a low SYNTAX Score II was compared with a higher score, and significantly higher predictive values for mortality were observed indicating that this new angiographic tool might be more effective to predict prognosis in patients who underwent PCI. Good calibration of the SYNTAX Score II has been demonstrated.^[23]^

Even a retrospective study from a single-center registry demonstrating the potential utility of the SYNTAX Score II in patients with left main CAD showed that SYNTAX Score II allowed a better and individualized risk stratification of patients requiring coronary revascularization.^[24]^ An editorial publication was also in favor of the SYNTAX Score II.^[25]^ The author stated that the SYNTAX Score II had far more benefits and it was innovative. He also suggested that the SYNTAX Score II was a daring attempt in this new era, and stated that the SYNTAX II trial was completed in November 2015 and it would be interesting to know its outcome.

To further support these current results, in another study, the authors concluded that the SYNTAX Score II might be applicable to several types of patients with CAD such as stable CAD, ACS and other patients with complex CAD undergoing PCI.^[10,26]^ A prospective study^[27]^ further stated that this important decision-making tool should be used in patients with 3-vessel diseases and in the vast majority of patients that have been enrolled in the SYNTAX II trial.^[28]^ It should also be noted that the SYNTAX Score II might also be important in contrast-induced nephropathy.^[29]^

This is one of the first meta-analyses to assess the risk of mortality prediction using the SYNTAX Score II scoring system in patients who underwent PCI. Another novelty is the fact that this scoring system is new in Interventional Cardiology and should be promoted and applied in daily practice. Applying this scoring system might better predict mortality following coronary angioplasty as compared with other tools such as the SYNTAX Score I.

This analysis has certain limitations. Even though the total number of participants was adequate, the number of participants involved in each subgroup was limited, and this might have affected the result that was obtained. In addition, each study had a different follow-up period. However, we could not improve on this part because the number of studies was limited after classifying them according to the standard low versus high SYNTAX Score II. Another limitation was the fact that participants with different types of CADs (upper LMCAD, STEMI, and old patients with CAD) were altogether analyzed. Nevertheless, as this current analysis was related to Interventional Cardiology and PCI, this limitation might be considered a minor one and might not affect the results to a significant extent. Also, 1 study consisted of participants who were also implanted with bare metal stents (BMS), whereas all the other studies involved patients who were implanted with drug eluting stents (DES). Other cardiovascular drugs, and antiplatelet therapy and its duration were also ignored in this analysis, and this might also represent a limitation. Moreover, a minor asymmetry in the funnel plot indicated moderate publication bias across the studies that assessed all the endpoints and this could represent a minor limitation of this analysis.

## 5 Conclusion

Following PCI, the risk of mortality was higher in those patients with a high SYNTAX Score II. The SYNTAX Score II might be considered as an important tool to predict mortality in Interventional Cardiology. Future research should further explore the benefits of this tool.

## Author contributions

HY, LZ and CX were responsible for the conception and design, acquisition of data, analysis and interpretation of data, drafting the initial manuscript, and revising it critically for important intellectual content. HY and LZ wrote this manuscript.

**Conceptualization:** Hua Yang, Li Zhang, Chen Hong Xu.

**Data curation:** Hua Yang, Li Zhang, Chen Hong Xu.

**Formal analysis:** Hua Yang, Li Zhang, Chen Hong Xu.

**Funding acquisition:** Hua Yang, Li Zhang, Chen Hong Xu.

**Investigation:** Hua Yang, Li Zhang, Chen Hong Xu.

**Methodology:** Hua Yang, Li Zhang, Chen Hong Xu.

**Project administration:** Hua Yang, Li Zhang, Chen Hong Xu.

**Resources:** Hua Yang, Li Zhang, Chen Hong Xu.

**Software:** Hua Yang, Li Zhang, Chen Hong Xu.

**Supervision:** Hua Yang, Li Zhang, Chen Hong Xu.

**Validation:** Hua Yang, Li Zhang, Chen Hong Xu.

**Visualization:** Hua Yang, Li Zhang, Chen Hong Xu.

**Writing – original draft:** Hua Yang, Li Zhang.

**Writing – review & editing:** Hua Yang, Li Zhang.
